# Biochemical characterization and mercury methylation capacity of *Geobacter sulfurreducens* biofilms grown in media containing iron hydroxide or fumarate

**DOI:** 10.1016/j.bioflm.2023.100144

**Published:** 2023-07-29

**Authors:** Elena Yunda, Quynh Nhu Phan Le, Erik Björn, Madeleine Ramstedt

**Affiliations:** Department of Chemistry, Umeå University, Sweden

## Abstract

*Geobacter* species are common in iron-rich environments and can contribute to formation of methylmercury (MeHg), a neurotoxic compound with high bioaccumulation potential formed as a result of bacterial and archaeal physiological activity. *Geobacter sulfurreducens* can utilize various electron acceptors for growth including iron hydroxides or fumarate. However, it remains poorly understood how the growth on these compounds affects physiological properties of bacterial cells in biofilms, including the capacity to produce MeHg. The purpose of this study was to determine changes in the biochemical composition of *G. sulfurreducens* during biofilm cultivation in media containing iron hydroxide or fumarate, and to quantify mercury (Hg) methylation capacity of the formed biofilms. Biofilms were characterized by Fourier-transform infrared spectroscopy in the attenuated total reflection mode (ATR-FTIR), Resonance Raman spectroscopy and confocal laser scanning microscopy. MeHg formation was quantified by mass spectrometry after incubation of biofilms with 100 nM Hg. The results of ATR-FTIR experiments showed that in presence of fumarate, *G. sulfurreducens* biofilm formation was accompanied by variation in content of the energy-reserve polymer glycogen over time, which could be cancelled by the addition of supplementary nutrients (yeast extract). In contrast, biofilms cultivated on Fe(III) hydroxide did not accumulate glycogen. The ATR-FTIR results further suggested that Fe(III) hydroxide surfaces bind cells via phosphate and carboxylate groups of bacteria that form complexes with iron. Furthermore, biofilms grown on Fe(III) hydroxide had higher fraction of oxidized cytochromes and produced two to three times less biomass compared to conditions with fumarate. Normalized to biofilm volume, the content of MeHg was similar in assays with biofilms grown on Fe(III) hydroxide and on fumarate (with yeast extract and without). These results suggest that *G. sulfurreducens* biofilms produce MeHg irrespectively from glycogen content and cytochrome redox state in the cells, and warrant further investigation of the mechanisms controlling this process.

## 1 Introduction

Bacteria have adapted to use various substances to gain energy for growth, and their growth and life cycle affect all biogeochemical processes on the planet [1]. Iron (Fe) reduction represents a significant route of bacterial respiration in soils, freshwater sediments, and anoxic aquifers [2]. In these environments, the majority of bacteria live in biofilms, and members of *Geobacter* genus are generally abundant [3]. *Geobacter* species can grow using iron- or manganese-based minerals as terminal electron acceptors, which are reduced through bacterial surface appendages and redox-active proteins, cytochromes [4,5]. Fumarate is an alternative electron acceptor in *Geobacter* respiration that is, in contrast to Fe(III), reduced intracellularly instead of extracellularly. Studies on the model organism, *G. sulfurreducens,* have shown that both Fe(III) and fumarate are suitable electron acceptors for biofilm formation by this species [[4], [5], [6], [7], [8], [9], [10]]. However, it is still not fully understood how the processes of biofilm growth, cell composition and metabolic features in biofilms are affected by growing *G. sulfurreducens* on these two different electron acceptors.

Owing to the ability to reduce Fe(III) hydroxides that are abundant in the environment, *Geobacter* species play significant roles in the geochemical cycling of many elements, including mercury (Hg). Thus, *G. sulfurreducens* is an important and widely used model to study biogeochemical transformations of Hg [[11], [12], [13], [14], [15], [16], [17]]. Mercury is one of the most hazardous elements present in aquatic systems and accumulated in biota. Through the consumption of seafood, humans can be exposed to high levels of methylmercury (MeHg) leading to neurotoxic effects with long-lasting consequences [18]. The transformation of inorganic divalent Hg (Hg(II)) to MeHg occurs due to physiological activity of diverse bacteria and archaea that all contain a gene pair encoding enzymes that carry out Hg methylation [19,20]. *Geobacter* species is one important group of Hg(II) methylators in the environment including in natural periphytic biofilms [21], and *G. sulfurreducens* has been used as a model in studies of environmental and biological factors controlling Hg(II) methylation [[11], [12], [13], [14], [15]]. However, there are no studies of MeHg formation by *G. sulfurreducens* biofilms cultivated on Fe(III) hydroxides despite the environmental relevance of these systems, and overall, the research on Hg(II) methylation in biofilms is still scarce [[22], [23], [24], [25]].

In this work, we study how several biofilm processes are affected by growing *G. sulfurreducens* on two different electron acceptors, i.e. Fe(III) hydroxide and fumarate. Fe(III) hydroxide represents an electron acceptor common in the natural environment, whereas fumarate is a standard electron acceptor used for cultivation of *G*. *sulfurreducens* [3,26]. First, we monitor differences in biochemical composition of *G. sulfurreducens* biofilms during their growth using a real-time and non-destructive Fourier-transform infrared spectroscopy in the attenuated total reflection mode (ATR-FTIR). ATR-FTIR measurements are further complemented by characterizing cultivated biofilms using Resonance Raman spectroscopy, highly sensitive to the redox state of cytochromes. Cytochromes play a key role in the interaction of *G*. *sulfurreducens* with extracellular electron acceptors and their characterization is important for a better understanding of biofilms differences dependent on the type of the electron acceptor used during their growth. Finally, biofilms cultivated in different conditions are studied in Hg(II) methylation assays under equal minimal nutritive conditions.

## 2 Materials and methods

### 2.1 Bacterial culture

*Geobacter sulfurreducens* PCA (ATCC 51573) was purchased from DSMZ and used in all experiments. The growth of bacterial culture was continuously maintained under N_2_ atmosphere at 30 °C and pH 6.8 in a growth medium adapted from Ref. [16] (Table SI1). For biofilm experiments, bacterial cultures at late-exponential growth phase were harvested by centrifugation (4000 rpm, 8 min) and resuspended at an optical density OD_660_∼0.02 in a medium used for biofilm formation experiments.

### 2.2 Bacterial suspensions for biofilm experiments

Bacterial suspensions for biofilm experiments were prepared in three conditions, in a medium containing (i) Fe(III) hydroxide as a sole electron acceptor, or in a medium containing fumarate, either (ii) with or (iii) without yeast extract. The detailed composition of the corresponding media is given in Table SI1 [16]. Fe(III) oxide was synthesized using the method of Schwertmann and Cornell [27]. Briefly, 100 mL of 0.2 M Fe(NO_3_)_3_ was titrated with 1 M NaOH to pH 7.5, followed by washing steps (centrifugation for 5 min at 8000 rpm and decantation) until reaching conductivity of ∼3.8 μS/cm. The structure of 2-line ferrihydrite was confirmed using X-ray diffraction analysis (data not shown). The product was freeze-dried and stored under N_2_ atmosphere.

### 2.3 Biofilm growth conditions

Glass cover slips (Menzel- Gläser, cut to a size 10 × 40 mm) were used as surfaces for biofilm growth for all experiments, except ATR-FTIR. A Zinc selenide (ZnSe) crystal (Harrick Scientific Products, US) with the dimensions of 20 × 10 mm (where one side includes a circle protrusion of ⌀17 mm serving as a substrate surface, Figure SI1) was used for ATR-FTIR experiments. When biofilms were studied on Fe(III) hydroxide, substrates surface area was coated with Fe(III) hydroxide suspension using drop-casting. Several tests with a range of suspensions from 0.2 to 1 g/L showed that 200 μL of 0.2 g/L suspension provided a stable film of Fe(III) hydroxide on the ZnSe crystal, sufficiently thin for IR beam to penetrate into the sample of bacterial biofilm. For glass substrates, 600 μL of 1 g/L Fe(III) hydroxide suspension was applied, as there was no constraint associated with the penetration depth and the surface area of glass slides was larger than the one of the ZnSe crystal. Bacterial cells were inoculated into 40 mL of the nutritive medium using N_2_-purged syringes or in a glove box with N_2_ atmosphere. The ZnSe crystal was enclosed in a custom-built ATR-FTIR measurement cell [28], whereas the glass substrates were kept horizontally in 50 mL anaerobic vessels. The substrates were cleaned and sterilized using Milli-Q water and 70% ethanol.

### 2.4 Monitoring of biofilm growth on ZnSe crystal *in situ* using ATR-FTIR

After bacterial inoculation, the ATR-FTIR measurement cell containing bacterial suspension was carefully installed in the FT-IR spectrometer (Vertex 80v, Bruker). The headspace of the ATR-FTIR cell containing a bacterial suspension was maintained under continuous N_2_ flow (0.05 bar) during spectral acquisition. During the first 3 h of the measurements, we observed fluctuations in bands corresponding to water. To allow for a stable background, the reference spectrum was recorded after 3 h of bacterial inoculation, after which the spectra were recorded every 20 min for the subsequent 69 h to monitor biofilm formation at static conditions in the room conditioned at 25 °C. Spectra were recorded on at least two sample replicates. ATR-FTIR spectra were recorded between 4000 and 700 cm^−1^. The resolution of the single beam spectra was 4 cm^−1^. A number of 100 scans were collected per spectrum. Spectra in the ATR mode are shown with an absorbance scale corresponding to log (R_reference_/R_sample_), where R is the internal reflectance. Spectra were recorded and processed for water vapor correction in OPUS 7.8. Integrated intensity calculations and baseline correction were performed with no preliminary spectral scaling using Matlab 2021a. Baseline was corrected as a straight line by setting at zero spectral intensities at 1800 and 900 cm^−1^, or 1800 and 800 cm^−1^, for conditions with fumarate or Fe(III) hydroxide, respectively. Integrated intensities were calculated to the obtained baseline.

### 2.5 Biofilm analysis using Resonance Raman spectroscopy

Biofilms prepared on glass substrates were analyzed using Renishaw Qontor Raman spectrometer, equipped with Leica microscope 100× objective and a 405 nm laser (40 mW) to capture a spectral range of 100–2300 cm^−1^. The analysis was performed in ambient air directly after harvesting the biofilms from the culture vessels and rinsing with 20 mM NaClO_4_. Only the first layer of cells in contact with the substrate surface was analyzed to ensure reproducible spectra of bacteria fully immersed in a medium. The spectral region around 900-950 cm^−1^ is susceptible to interferences from glass substrates, and it was therefore omitted from spectral interpretation. Data acquisition was performed using raster scanning with 100% intensity laser power with exposure time of 1 s. Five random areas in biofilms were selected for spectral acquisition. All spectral analyses and data processing were performed using Renishaw WiRE 5.3 software. The experiments were performed on at least two sample replicates. Sample of cytochrome *c* protein from horse heart (Sigma, St. Louis, MO) was used for comparison with biofilm spectra. The protein (5 μM) was dissolved in 0.1 M phosphate buffer at pH = 7.0. Immediately before Resonance Raman measurements, the proteins were reduced by the addition of an excess amount of sodium dithionate (equiv 1:10).

### 2.6 Quantification of biofilm volume

Biofilms prepared on glass substrates were examined using Nikon A1R confocal microscope and the volume was estimated using BiofilmQ software, as previously described [23,29]. Briefly, biofilms were stained with Syto9 dye (Invitrogen) in dark for 15 min and imaged every 1.74 μm along the biofilm height using a DIC 20× objective. Series of images were recorded on a minimum of three replicate samples. The total volume of biofilms in the assay was then calculated by extrapolating the value obtained for the image to the total surface area of one side of the glass slide. Thus, the data presented assumes that glass slides were fully covered with biofilms and that the obtained images are representative enough to allow extrapolation to the total surface area of the glass substrates.

### 2.7 Exposure of biofilms to Hg(II) and quantification of MeHg

The detailed protocol of Hg(II) methylation assay with *G. sulfurreducens* biofilms is described elsewhere [23]. That protocol was used here with some minor adjustments, as follows. Biofilms were cultivated on glass cover slips for 72 h under conditions (i), (ii) or (iii) as described above and then transferred into an assay buffer with 100 nM Hg(II) (Hg(NO_3_)_2_ stock solution, 28,941-100 ML-F, 191, Fluka). The volume of the assay buffer was 6 mL. The composition of the assay buffer for all biofilms contained 1 mM acetate, 1 mM fumarate, 10 mM MOPS, 0.1 mM NH_4_Cl, 1.3 mM KCl, 0.15 mM MgSO_4_, 5 mM NaH_2_PO_4_, 0.17 mM NaCl, and 1 mg/L resazurin [17]. This assay buffer allows supporting cell metabolic activity without substantial cell growth, and it has been previously used in studies reporting Hg(II) methylation assays with *G*. *sulfurreducens* [12,16,17,23]. MeHg formed in the assay was quantified by isotope dilution analysis using Me^200^Hg (synthesized in house according to Snell et al. [30]). After incubation with Hg(II), Me^200^Hg was added in the vessels containing biofilms followed by addition 1:10 (v/v) of 25% (w/v) NaOH. Vessels were vigorously shaken and left in static conditions for 24 h at room temperature. The obtained samples contained the sum of MeHg associated with biofilms and MeHg released in the biofilm-surrounding medium during the assay. Thermal desorption gas chromatography combined with inductively coupled plasma-mass spectrometry (TD-100 Markes – GC 7890 B Agilent – ICP-MS 7700 Agilent) were used to determine MeHg concentration in samples. Prior to analysis, samples were pH adjusted to 4.5 using 5 M HCl and 2 M CH_3_COONH_4_, derivatized with NaB(C_2_H_5_)_4_ and purged with N_2_ onto Tenax adsorbent tubes. Statistical analysis was performed using Student's t-test function of Microsoft Excel software.

In separate experiments, total Hg concentrations were determined in vessels after 6 h of incubation with biofilms. Triplicate samples for each condition were spiked with a ^200^Hg standard (96.41% enrichement, batch 185,091, Oak Ridge National Laboratory, TN, USA) and digested with BrCl following the EPA 1631 E method [31]. The spiking and digestion steps were performed in the same vials where the incubation of the biofilms with Hg(II) was carried out, immediately after the end of the assay. Samples were neutralized with NH_2_OH·HCl and analyzed using a CETAC HGX-200 cold vapor system coupled with ICPMS (8900 Agilent) after online Hg(II) reduction with SnCl_2_.

## 3 Results

### 3.1 Monitoring biofilm chemical composition in conditions with fumarate

**Fig. 1:**
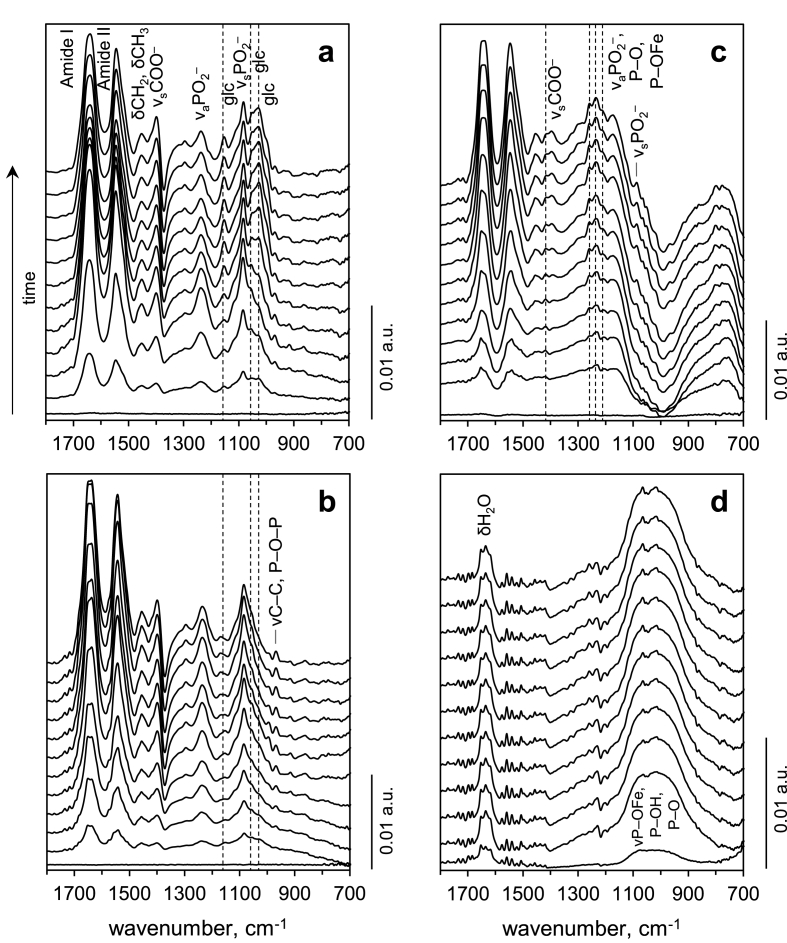
Time evolution of ATR-FTIR spectra during biofilm formation by *G*. *sulfurreducens* in the medium with a) fumarate, b) fumarate and yeast extract, or c) Fe(III) hydroxide. d) Spectra of the nutritive medium lacking fumarate and lacking bacterial cells, recorded on Fe(III) hydroxide. Spectra in a-d) are offsets corresponding to 3, 9, 15, 21, 27, 33, 39, 45, 51, 57, 63, and 69 h after the start of the measurements, from bottom to top respectively. Reference spectra were obtained after 3 h of inoculation of a-c) bacterial suspensions or d) nutritive medium.

Bacterial aggregation and surface attachment are the first steps of biofilm formation [32]. Here, we apply a surface-sensitive and non-destructive approach to monitor biofilm development and changes in its chemical composition over time. First, we studied biofilm formation in the medium containing fumarate, a standard electron acceptor used in research on *G. sulfurreducens*. Bacterial cells were inoculated in the measurement cell for ATR-FTIR measurements and spectra were recorded continuously for 3 days to follow cell attachment and evolution of biofilm chemistry (Fig. 1a).

The spectra contain information on the fingerprint region represented mainly by proteins (1690–1530 cm^−1^), polysaccharides (1200–900 cm^−1^), and a mixture of nucleic acids and phospholipids (1240–1220 cm^−1^) [33,34]. The increase of the spectral regions corresponding to main cellular components indicates presence of increasing number of bacterial cells in contact with the substrate surface and biofilm development. The increase of bacterial bands was accompanied by the decrease of the band at 1373 cm^−1^ assigned to COO^−^ stretching vibrations of fumarate (Figure SI2). *G*. *sulfurreducens* converts fumarate to succinate during its growth [35], and the accumulation of succinate was suggested by its bands at ∼1550, 1396 and 1296 cm^−1^ in the spectrum of metabolites collected from the medium after biofilm cultivation (Figure SI2) [36]. As the biofilms developed over time, several bands in the polysaccharide region changed remarkably. These bands, at 1155, 1048, and 1025 cm^−1^, were assigned to glycogen, an energy-reserve polymer [37]. Glycogen bands were present at the initial stage of the biofilm growth, and their intensity decreased until ∼20 h. After this time point, the intensity of glycogen bands progressively increased. As glycogen storage is primarily intracellular [38], the variation in the intensity of the corresponding bands suggests utilization and subsequent build-up of this energy source in bacteria. Indeed, we compared the spectra of biofilms with those of planktonic cell cultures and found similar evolution of glycogen bands during 48 h (Figure SI3).

**Fig. 2:**
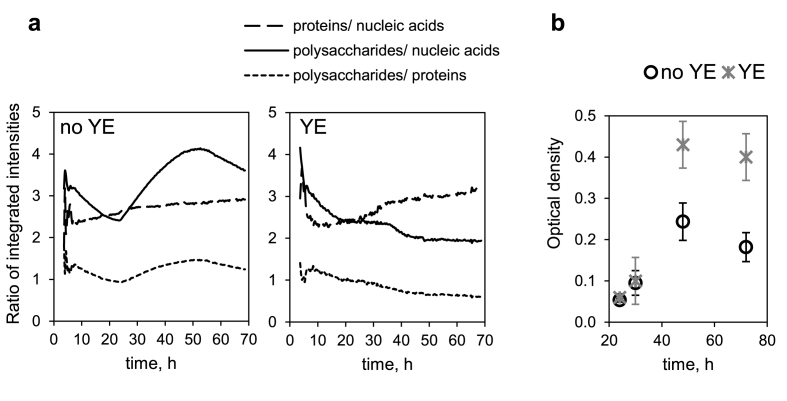
A) Ratios of integrated intensities of spectral regions corresponding mainly to proteins, nucleic acids and polysaccharides plotted as a function of time during biofilm growth of *G*. *sulfurreducens*. Proteins: integration between 1597 and 1483 cm^−1^ corresponding to amide II band; nucleic acids: 1280–1188 cm^−1^; polysaccharides: 1187–952 cm^−1^. YE – yeast extract added in the nutritive medium for biofilm growth. Please note that the molar absorption coefficient between vibrations in macromolecules are different from each other. b) Growth of *G. sulfurreducens* in planktonic cultures with or without yeast extract in the nutritive medium.

Since the ability to synthesize glycogen is linked to the nutrient availability [39], we investigated how supplementary nutrient load would impact this metabolic feature. Fig. 1b shows spectral evolution of the biofilm cultivated in the medium where yeast extract was added. Glycogen was not accumulated in the biofilm in nutrient-rich conditions, while synthesis of other cellular compounds (i.e. proteins and nucleic acids/phospholipids) remained high. Therefore, the relative content of polysaccharides was eventually lower in the biofilm cultivated in nutrient-rich conditions. This can be further demonstrated by the time evolution of ratios of integrated intensities of bands corresponding to proteins, nucleic acids and polysaccharides (Fig. 2a). For proteins, amide I and amide II bands are potentially influenced by H_2_O bending and fumarate/metabolite COO^−^ asymmetric stretching vibrations, respectively (figure SI2). The amide II band was selected for the integration analysis under assumption that the interference from fumarate COO^−^ band decrease is compensated by metabolite COO^−^ band increase, and that these effects are similar in conditions with and without yeast extract. The ratios of proteins to nucleic acids remained similar in conditions with and without yeast extract. Consequently, from the continuous decrease of ratios polysaccharides/proteins and polysaccharides/nucleic acids during biofilm formation in medium with yeast extract, it can be concluded that proteins and nucleic acids were synthesized at a higher rate than polysaccharides during the whole period of biofilm formation in this more nutrient rich medium. The same trend was observed for biofilm grown in fumarate medium without yeast extract for the first 24 h. However, following this time point the rate of polysaccharide formation was higher, as indicated by the increase in ratios polysaccharides/proteins and polysaccharides/nucleic acids.

It is interesting to note that in planktonic cell cultures in the nutritive media with fumarate, cells begin multiplication after approximately 24 h, and this process is slower in absence of the yeast extract (Fig. 2b). The ATR-FTIR results suggest that the lower biomass production is associated with the metabolism directed towards polysaccharide synthesis.

### 3.2 Monitoring bacterial attachment and biofilm growth on Fe(III) hydroxide

Further ATR-FTIR experiments were performed using Fe(III)-coated substrates and in absence of fumarate and yeast extract in the nutritive medium. Progressive bacterial attachment and biofilm formation on the Fe(III) surface was observed, and is seen in the spectra as increasing intensities of protein bands throughout the measurement (Fig. 1c), similar to observations on bare ZnSe. However, the remaining part of the fingerprint region looks remarkably different from the conditions with fumarate (Fig. 1a and b). First, negative intensities are seen in the region at 1150–800 cm^−1^ (note that each spectrum has been base line corrected to 0 absorption units at wavenumber 800 cm^−1^, thus, the low intensity between 1150 and 800 cm^−1^ represents negative absorption values). This region comprises a number of bands corresponding to phosphate groups and phosphate complexes with iron. The bands at ∼980, 1000, 1025 and 1040 cm^−1^ are assigned to a mixture of P–OFe, P–OH and P–O stretching vibrations, while the band at ∼1120 cm^−1^ is assigned to P=O stretching vibrations [40,41]. Phosphate/phosphonate functional groups are present in the bacterial cell wall in phospholipids and lipopolysaccharides [42], but phosphate is also present in the nutritive medium. To elucidate the respective contribution of phosphates present in the nutritive medium and in the bacterial cell wall, spectra of pure nutritive medium were recorded on the Fe(III) hydroxide surface (Fig. 1d). These spectra showed pronounced bands between 1150 and 800 cm^−1^, the shape of which corresponded well to the shape of the negative bands in Fig. 1c. This result suggests that phosphates, which had initially been present in the nutritive medium and adsorbed onto the Fe(III) hydroxide surface, were progressively removed during biofilm formation. Spectra recorded during biofilm formation (Fig. 1c) at a similar time frame, showed a concomitant increase of several bands with peak maxima at 1260, 1234, 1211, 1066, and 1018 cm^−1^ corresponding to phosphate complexes with iron [41,43]. The rise of these bands was accompanied by the relative increase in proteins and other typical bacterial functional groups (e.g. bands at 1450, 1400, and ∼1090 cm^−1^ (Fig. 1c), indicating cell growth on the Fe(III) hydroxide surface. Thus, it can be concluded that phosphate from the nutritive medium that had adsorbed on the Fe(III) hydroxide surface was gradually replaced by the phosphate/phosphonate functional groups present on the bacterial cell wall.

A second notable difference, from spectra acquired in fumarate conditions, was a shift of both PO_2_^−^ bands in the region 1250–900 cm^−1^, and COO^−^ vibrations around 1400 cm^−1^. PO_2_^−^ stretching vibrations shifted from 1234, 1088, and 967 cm^−1^ to 1260, 1211, 1083 and 972 cm^−1^, while carboxyl groups shifted from 1400 cm^−1^ to 1418 cm^−1^. The peak shift of COO^−^ stretching vibration is indicative of the formation of carboxylate-Fe(III) complexes [43]. Similarly to phosphates, carboxyl groups can be found on the bacterial surface, for example in the amino acids of membrane proteins. Thus, these results show that the adhesion and biofilm formation of *G. sulfurreducens*s are mediated to some extent by the interaction of carboxylate and phosphate groups with the Fe(III) hydroxide surface.

Since spectral features of the biofilm formed on Fe(III) hydroxide were complicated by the nutritive medium contribution and binding to Fe(III) hydroxide surface, we did not compare the time evolution of ratios of integrated intensities of bands corresponding to proteins, nucleic acids and polysaccharides with those obtained in fumarate conditions. Nonetheless, the second derivatives of the spectra allowed us to obtain details on the spectral differences between Fe(III) hydroxide and fumarate conditions (Figure SI4). As it is suggested by the lack of the band at 1155 cm^−1^, there was no glycogen accumulation in the biofilm formed on Fe(III) hydroxide.

### 3.3 Characterization of cultivated biofilms using Resonance Raman spectroscopy

**Fig. 3:**
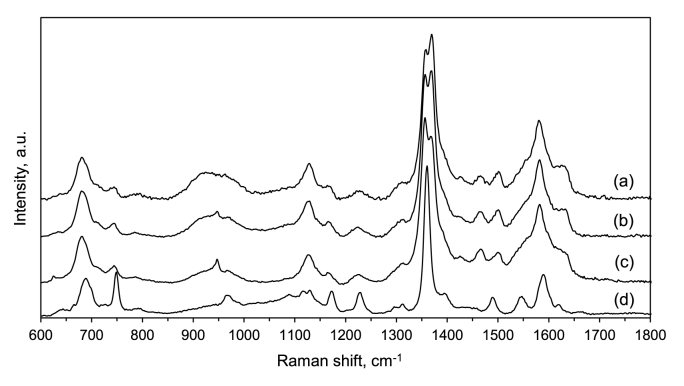
Raman spectra of the first bacterial layer of biofilms grown on glass substrates in three different nutritive media: a) lacking fumarate and containing Fe(III) hydroxide as a coating on the glass surface, b) containing fumarate, and c) containing fumarate and yeast extract; d) Raman spectrum of cytochrome *c* from horse heart in the reduced state. All spectra are normalized to the maximum intensity, offsets are used for clarity.

Next, we characterized biofilms using Resonance Raman spectroscopy after 72 h of growth. The wavelength of the excitation laser line (405 nm) was in proximity to an electronic transition of the heme group in a cytochrome *c*, resulting in the enhancement of bands corresponding to cytochrome *c* in the spectra. Thus, the Resonance Raman spectra presented in Fig. 3 contain mainly information on the chemistry of cytochrome *c* in *G. sulfurreducens* biofilms. The spectra of biofilms were compared with the spectrum of commercial cytochrome *c* from horse heart. A detailed band assignment is given in Table SI2 and Figure SI5 [44]. Compared to cytochrome *c* from horse heart, the Resonance Raman spectra of *G. sulfurreducens* cytochrome *c* in the biofilms exhibit considerable shift and broadening in several bands. A shift can be seen in the band at 1582 cm^−1^ corresponding to C_α_–C_m_ antisymmetric stretching vibrations of the porphyrin ring, the band at 743 cm^−1^ related to pyrrole stretching vibrations, and the bands at 681 cm^−1^ corresponding to C–S stretching, while the band at 1225 cm^−1^ assigned to C_m_–H bending was broadened. These differences could be associated with the fact that *G. sulfurreducens* contains over 100 cytochromes, many of which are multiheme [45], in contrast to horse heart cytochrome *c* that has a single heme. Furthermore, in biofilms cytochrome *c* is dynamic and can exist in various forms in contrast to static conditions of cytochrome *c* in the reference.

The spectral profiles of *G. sulfurreducens* biofilms obtained in three nutritive conditions were overall similar, suggesting presence of cytochrome *c* in all biofilms. However, there was a notable variation in biofilm spectral features in the 1350–1370 cm^−1^ region, which is sensitive to cytochrome redox state. The bands in this region correspond primarily to C–N stretching vibrations of porphyrin rings (figure SI5) [46]. In the reduced state of the cytochrome, the C–N bond is weakened due to a greater overlap between electron orbitals of the porphyrin ring and Fe(II) compared to Fe(III), resulting in the shift of C–N band to a lower wavenumber (∼1360 cm^−1^) [47]. In the oxidized state of the cytochrome, this band is shifted towards higher wavenumber and is localized at ∼1370 cm^−1^. The biofilm grown on Fe(III) was characterized by spectra showing cytochrome *c* predominantly in the oxidized state as the peak intensity at 1370 cm^−1^ was higher than that at 1357 cm^−1^ (Fig. 3). This is also supported by a higher intensity of the bands corresponding to C_α_–C_m_ porphyrin symmetric and antisymmetric stretching vibrations at 1496 cm^−1^ and 1634 cm^−1^, respectively, which are more intense in the oxidized state of cytochrome *c* [48]. In conditions with fumarate, and more pronounced in the medium with yeast extract, cytochromes in the reduced state were more abundant than in the biofilms grown on Fe(III) hydroxide.

### 3.4 Exposure of biofilms to Hg(II) and MeHg formation

*G*. *sulfurreducens* is a common model to study biological and chemical factors controlling Hg transformations, such as MeHg formation in assays with fumarate-grown bacterial cultures [11,12,[15], [16], [17]]. We studied how biofilm cultivation on Fe(III) hydroxide may influence MeHg production in comparison with fumarate-grown biofilms. In further experiments, biofilms cultivated for 72 h in conditions with Fe(III) hydroxide or fumarate with or without yeast extract were incubated in an assay medium (described in *Materials and methods*) with 100 nM Hg(II) for additional 0–48 h.

**Fig. 4:**
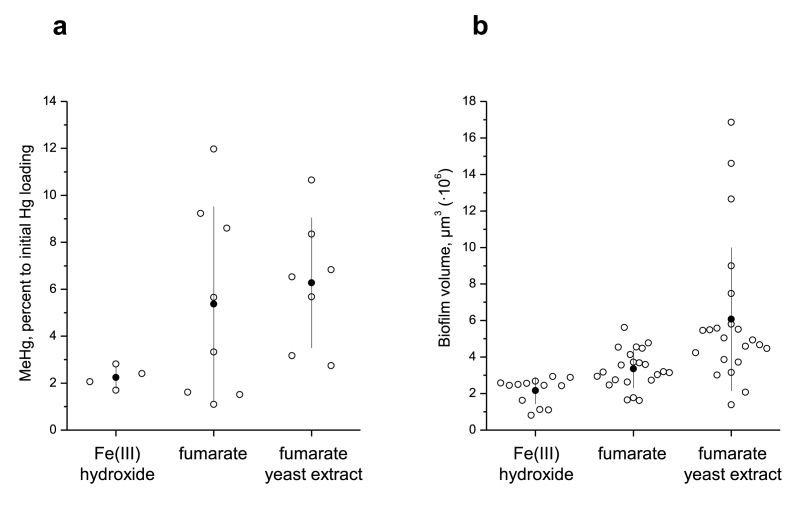
A) Percentage of MeHg produced from 100 nM added Hg, determined after 6 h of incubation with *G*. *sulfurreducens* biofilms and b) volume of biofilms grown for 3 days in nutritive media containing Fe(III) hydroxide or fumarate, with or without yeast extract. Empty and filled circles represent replicates and average values, respectively. Vertical lines correspond to standard deviations, the spread along the X axis within the category is to improve visibility. The volume corresponds to the area of images obtained using 20× objective (0.41 mm^2^).

We observed MeHg formation by biofilms cultivated both on Fe(III) hydroxide and fumarate, with similar time evolution of MeHg formation (Figure SI6). After approximately 6 h of biofilm incubation with Hg(II), there was no longer any significant increase in MeHg concentration, while the recovery of total Hg was 94 ± 3% and not different among treatments. Since the majority of MeHg was produced within the first 6 h further quantification of MeHg production was performed in 6-h assays. Compared to conditions with fumarate, biofilms grown on Fe(III) hydroxide, on average, methylated around three times less Hg(II) than biofilms grown in fumarate conditions with and without yeast extract; 2.2% vs. 6.3% and 5.4% of the added Hg(II), respectively (Fig. 4a). This difference was significant for biofilms grown with but not without yeast extract (p = 0.008 and p = 0.07, respectively). To normalize MeHg produced in the assays to biofilm content, confocal microscopy images of biofilms were used to estimate biofilm volume after growth in each nutritive condition; the images representative for biofilms of average found volume are shown in Figure SI7. As shown in Fig. 4b, biofilms grown on Fe(III) hydroxide had approximately three and two times less volume than biofilms grown in medium with fumarate with and without yeast extract. The average values of MeHg produced per biofilm volume were found to be 1.1 × 10^−18^, 1.0 × 10^−18^, and 1.6 × 10^−18^ M/μm^3^ for biofilms grown on Fe(III) hydroxide and fumarate with and without yeast extract, respectively. Thus, no difference in methylation capacity was observed when values were normalized to biofilm volume.

## 4 Discussion

This study contributes to a continuous general effort to decipher the link between nutrient conditions and bacterial behavior in biofilms [49,50]. Fumarate and Fe(III) (as a soluble citrate or solid oxide) are two conditions that have already been used in parallel in studies of *G. sulfurreducens* biofilms [4,5]. These studies addressed the properties and role of *G. sulfurreducens* pili. However, the majority of research including these two conditions has been performed on planktonic cultures and focused on metabolic features of *G. sulfurreducens* [35,[51], [52], [53], [54], [55], [56]]. Our study provides further insight into the mechanisms of *G. sulfurreducens* adaptation to different nutritive media using *in situ* ATR-FTIR monitoring of molecular fingerprints of biofilms. Previous research has proven ATR-FTIR spectroscopy to be useful for non-destructive analysis of the effect of nutrients on biofilm development [57,58]. For example, a change in accumulation of biofilm polysaccharides and other metabolic products as a function of nutritive medium selected for biofilm growth was shown in a study of *Lactobacillus rhamnosus* [58]. Furthermore, FTIR spectroscopy is particularly sensitive to energy storage compounds synthesized by bacterial cells, such as polyhydroxyalkanoates [59] and glycogen [37].

A possible role of glycogen as a long-term electron storage in electroactive *G. sulfurreducens* biofilms has been addressed [60]. The evidence of glycogen accumulation in *G. sulfurreducens* obtained using ATR-FTIR spectroscopy has recently been reported [61]. It was found that upon the transfer of planktonic cells into a minimal nutritive medium used for Hg(II) methylation assays the content of glycogen in bacteria varies as a function of time. Glycogen provides a number of benefits to the cells including higher chances of survival and faster adaptation to fluctuating environments [39]. The results of our study indicated glycogen decrease in the first 20 h of biofilm growth, a period corresponding to the typical lag phase in planktonic cultures, followed by glycogen increase until the moment when stationary phase is usually reached. In a study on *Escherichia coli* [62], the period of glycogen depletion also corresponded to a lag phase of bacterial growth, whereas the period of glycogen synthesis was associated with cell division. These and other metabolic profile data allowed the authors to conclude that glycogen is a primary energy source for *E*. *coli* cells when they encounter resupply of nutrients after exhaustion. The function of glycogen was thereafter interpreted as an ‘energy bank’ from where resources are withdrawn to build the machinery for nutrient uptake and utilization, and to where the energy is deposited once the metabolism in a fresh nutritive medium is well established [39,63]. The results obtained on *G. sulfurreducens* in the present study support this notion.

Furthermore, glycogen is generally synthesized when there is excess of carbon relatively to other nutritive elements in the medium [64]. The absence of glycogen accumulation in presence of yeast extract can therefore be explained by the increase in nitrogen and phosphorus content present in the undefined mixture of amino acids, peptides and vitamins in the yeast extract. It is also interesting to note that fumarate was suggested to serve as a complementary carbon source in addition to acetate in *G. sulfurreducens* cultures [51]. The use of available fumarate for building glycogen storage could therefore explain its presence in biofilms in fumarate conditions and its absence in conditions with Fe(III) hydroxide.

The ATR-FTIR spectra of *G. sulfurreducens* biofilms cultivated on Fe(III) hydroxide revealed details in the molecular interactions between bacterial cells and the Fe(III) hydroxide surface. It should be noted that, generally, at pH around 7 bacteria are negatively charged, including *G. sulfurreducens* [65]. Iron oxide surface has a positive charge in this pH range and therefore, bacterial adhesion is electrostatically favored [66]. However, the spectral features of biofilms obtained on iron oxide coating indicated that the interactions were more complex than only electrostatic attraction. Formation of bonds between bacteria and iron was indicated by the appearance of bands attributed to iron complexes with phosphate-bearing molecules, as well as the shift of the band assigned to carboxylate groups. Thus, the results suggest that at least part of the surface interaction was due to formation of complexes between iron and carboxylate or phosphate moieties present, for example in cell surface proteins, phospholipids and lipopolysaccharides.

Formation of chemical bonds between *G. sulfurreducens* and iron is in accordance with previous ATR-FTIR studies of cell-mineral interactions on other bacteria common in soil, including *Pseudomonas* spp. [41,[66], [67], [68]], *Shewanella* spp. [41,69], and *Bacillus subtilis* [41,67,68]. In some of these studies, it was suggested that bacterial binding occurs primarily via phosphates, while carboxylate groups are involved less or at later stages [41,69]. Furthermore, using single-molecule force spectroscopy it was shown that the interaction between phosphate and iron oxide is stronger than that with carboxylate groups [70]. However, in our study, the shift in the band corresponding to carboxylate group was prominent already at the early stages of biofilm formation. This result could be explained by the presence of phosphate in the nutritive medium and its competition with phosphate groups of bacteria for iron binding. Indeed, during biofilm formation on iron oxide coatings we observed the increase of bacteria-associated bands in conjunction with the decrease of bands associated with phosphates from the nutritive medium. Desorption of phosphate from iron-coated surfaces during bacterial attachment was also found in a study on *Bacillus subtilis* and *Pseudomonas fluorescens* [67]. Furthermore, the pronounced role of carboxylate groups in attachment observed in our study could also be due to carboxylate-bearing molecules in the cell wall and biofilm matrix of *G. sulfurreducens*, such as cytochromes [71]. The surface of cytochrome *c* contains carboxylate groups [72], and cytochromes of *G. sulfurreducens* play critical role in bacterial contact with iron [73,74]. This mechanism of interaction with solids is distinct from those in other bacterial species. For example, it is known that *Pseudomonas* spp. mediate adhesion to surfaces via extracellular DNA [75,76]. In line with this knowledge, the ATR-FTIR study on extracted extracellular polymeric substances (EPS) of *Pseudomonas aeruginosa* showed a primary role of phosphodiester groups of nucleic acids in EPS binding to iron oxide [68]. A more in-depth study would be necessary to determine which bond dominates the adhesion of *G. sulfurreducens* onto iron oxides, but our study shows that both phosphate and carboxylate groups are involved in the attachment to Fe hydroxides.

A particular focus of our work was to investigate biofilm interactions with Fe(III) hydroxide, in which cytochromes are known to play an important role [74]. Cytochrome *c* of *G. sulfurreducens* biofilms grown on electrodes was previously characterized with Raman spectroscopy in a resonance mode [77,78]. Here, using this method we characterized biofilms grown on glass or iron oxide with and without fumarate in the medium, and observed the highest fraction of oxidized cytochrome *c* when biofilms were formed on the iron oxide surface. This observation is in accordance with the notion that cytochrome *c* acts as Fe(III) reductase during growth of *G. sulfurreducens* [79]. The result also agrees with Raman studies of electroactive *G. sulfurreducens* cells attached to electrodes. Indeed, the fractions of oxidized cytochromes in biofilms on electrodes set at potentials comparable to those of poorly crystalline iron oxides (∼0 V [80]) were notably high [77,78]. The lower fraction of oxidized cytochromes observed in fumarate conditions could be associated with fumarate being reduced in the intracellular compartment via metabolic pathways not involving cytochromes [81]. Furthermore, in biofilms obtained under fumarate conditions with the addition of yeast extract the majority of cytochromes were reduced. Yeast extract can serve as a potential electron donor [82], and studies on *G. sulfurreducens* planktonic cultures show that cytochrome *c* is reduced in conditions with electron donor excess [79,83]. Therefore, the results of our study are in good agreement with previous reports.

The influence of iron on microbial activity is important to consider from a biogeochemical perspective. There is a growing evidence of the significant contribution of iron-reducing bacteria in the process of MeHg formation in natural environments [21,84]. Since the discovery of Hg(II) methylation by iron-reducing bacteria [85] *G*. *sulfurreducens* has become one of the primary model species used in mechanistic studies of this process [11,12,[15], [16], [17]]. However, very few studies investigated how Hg(II) methylation by *G*. *sulfurreducens* occurs when cells are cultivated on Fe(III) instead of fumarate [13,86]. Here, it is worth noting that studies performed on bacterial isolates and aimed to determine the mechanisms of MeHg formation on a molecular scale require a very careful control of chemical and biological factors that could influence the assay. In the assays used here we have primarily focused on the possible influence of biochemical differences in biofilms grown in media with different electron acceptors on the process of MeHg formation. It should be noted that addition of Fe(III) hydroxide into the system may influence bioavailable Hg due to a possibility of Hg complexation by iron or Hg reduction by Fe(II) produced as a result of Fe(III) reduction [87]. Thus, in order to minimize the contribution from inorganic Fe(III) hydroxide on Hg bioavailability we designed our experiments in a way so that the mineral surface would be covered by biofilm before exposure to Hg.

We found lower overall formation of MeHg in assays with Fe(III)-grown biofilms. However, the determined biofilm volume suggests that the amount of MeHg produced is proportional to the biofilm content in the assay. Indeed, the biofilm volume on Fe(III) hydroxide was on average 2 to 3 times less than in conditions with fumarate. These data are in line with studies on *G. sulfurreducens* planktonic cultures reporting biomass yields being 3-fold lower in cultures with Fe(III) citrate compared to fumarate [35]. It has been suggested that electron transfer to Fe(III) is less efficient in terms of promoting biomass growth due to additional energy required to maintain cell proton balance once electrons are transported out of the cell [35]. Furthermore, fumarate can act as an additional carbon source enabling higher biomass production [51]. In this regard, our ATR-FTIR results on glycogen accumulation in fumarate–grown biofilms (in absence of yeast extract) are consistent with higher biomass yields obtained with fumarate than with Fe(III) hydroxide.

Normalized to biofilm volume, the values of MeHg were very similar in conditions with biofilms grown on Fe(III) hydroxide and on fumarate (with yeast extract and without). This result is noteworthy considering the multiple physiological adjustments cells need to undertake to adapt to different electron acceptors and that these adaptations previously have been described to affect biomass growth. However, the biological function of Hg methylation remains poorly understood. In assays with *G. sulfurreducens* planktonic cultures, it was found that MeHg does not accumulate inside bacterial cells, and a possibility of MeHg formation as bacterial detoxification mechanism was discussed [14]. We have previously shown that, for *G. sulfurreducens* biofilms [23], the accumulation of MeHg inside bacterial cells was also not significant while the capacity to produce MeHg was high, similar to the results in this present study. Furthermore, we observed that the rates of Hg(II) methylation by *G. sulfurreducens* biofilms were relatively similar to those of planktonic counterparts [23]. Altogether, these findings suggest that different *G. sulfurreducens* phenotypes possess similar capacity to methylate Hg(II), and warrant further investigation of the mechanisms controlling this process.

## CRediT authorship contribution statement

**Elena Yunda:** Conceptualization, Methodology, Investigation, Formal analysis, Writing – original draft, Writing – review & editing. **Quynh Nhu Phan Le:** Methodology, Investigation, Formal analysis, Writing – review & editing. **Erik Björn:** Conceptualization, Methodology, Formal analysis, Writing – review & editing, Funding acquisition. **Madeleine Ramstedt:** Conceptualization, Methodology, Formal analysis, Writing – review & editing, Funding acquisition.

## Declaration of competing interest

The authors declare that they have no known competing financial interests or personal relationships that could have appeared to influence the work reported in this paper.

## Data Availability

Data will be made available on request.
