# Supplementary material for: Biochemical characterization and mercury methylation capacity of Geobacter sulfurreducens biofilms grown in media containing iron hydroxide or fumarate
Source: Biofilm. 2023 Jul 29;6:100144. doi: 10.1016/j.bioflm.2023.100144 (PMC10424081; doi:10.1016/j.bioflm.2023.100144)
Supplement: Multimedia component 1 [file mmc1.docx]

**Supporting information**

Figure SI1. ZnSe crystal coated with Fe(III) hydroxide and ATR-FTIR accessory used for spectral acquisition during biofilm formation. Enclosed at the bottom of the accessory is the ZnSe crystal.


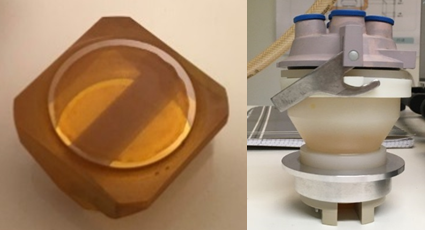


Figure SI2. Time evolution of ATR-FTIR spectra during biofilm formation by *G*. *sulfurreducens* in the medium with fumarate with or without yeast extract (YE), from bottom to top: 3, 9, 15, 21, 27, 33, 39, 45, 51, 57, 63, and 69 hours after the start of the measurements. The reference spectra for biofilm series were obtained by recording the medium with bacterial cells after 3 hours of inoculation of bacterial suspensions. The reference spectrum for sodium fumarate was MQ water. The spectrum of metabolites was obtained by taking the aliquot from the top part of the IR accessory after 72 hours of biofilm growth. The reference spectrum and the spectrum of metabolites were recorded just after the aliquot was placed, and after ~1 hour of aliquot contact with the crystal, respectively.

Figure SI3. ATR-FTIR spectra of *G*. *sulfurreducens* biofilms and planktonic cells recorded after the growth in the nutritive with fumarate. The spectra of planktonic cells were recorded by centrifuging cultures and placing collected pellets on the diamond ATR accessory, where supernatant was used as a reference spectrum. The spectra of biofilms and planktonic cells were normalized to the same intensity of Amide II band at 1548 cm^-1^.


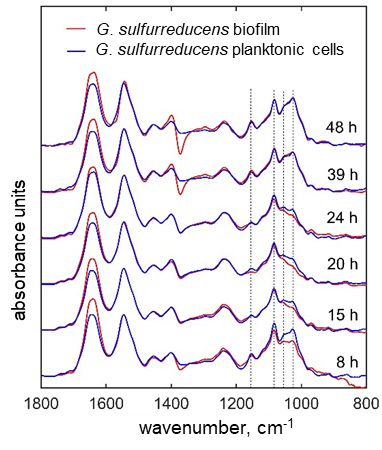


Figure SI4. ATR-FTIR spectra of *G*. *sulfurreducens* biofilms recorded after 69 hours of cultivation with a) fumarate or b) Fe(III) hydroxide in the medium, with c-d) corresponding second derivative spectra. The dash lines indicate glycogen bands found in conditions with fumarate and absent in conditions with Fe(III) hydroxide.

Figure SI5. Heme scheme of cytochrome *c* [1].

Figure SI6. MeHg formation in assays with biofilms obtained in media with a) Fe(III) hydroxide, b) fumarate, or c) fumarate and yeast extract. After the growth in respective media for 72 hours, biofilms were transferred in the assay medium with minimal nutrient load for incubation with 100 nM Hg(II) for 0–48 hours (circles). Control samples were included: without added Hg(II) (crosses) or without added biofilms and containing 100 nM Hg(II) (triangles). Each point represents one individual sample.

Figure SI7. Confocal laser scanning microscopy images of biofilms obtained in media with a) Fe(III) hydroxide, b) fumarate, or c) fumarate and yeast extract, corresponding to samples with biofilm volume close to the average among replicates. The scale bar is 50 µm.

Table SI1. Composition of nutritive media used for biofilm experiments

|  | **Fe(III)** | **Fumarate with yeast extract** | **Fumarate without yeast extract** |
| --- | --- | --- | --- |
| sodium fumarate, mM | – | 40 | 40 |
| yeast extract, g/L | – | 1 | – |
| Fe(III) hydroxide, µg/mm^2^ | ~0.2 (ZnSe); ~1.5 (glass) | – | – |
|  |  | | |
|  | **All treatments** | | |
| sodium acetate, mM | 10 | | |
| MOPS, mM | 10 | | |
| NH_4_Cl, mM | 5.6 | | |
| KCl, mM | 1.3 | | |
| NaCl, mM | 0.2 | | |
| MgSO_4_, mM | 0.1 | | |
| CaCl_2_, μM | 8.8 | | |
| NaH_2_PO_4_, mM | 0.05 | | |
| Na_2_SeO_3_, μM | 0.6 | | |
| Wolfe’s trace metals with 1:10 CuSO_4_ | 1% (v/v) | | |
| resazurin, mg/L | 1 | | |

Table SI2. Assignments of Raman spectra of cytochrome *c* [1]

| Raman shift (cm^-1^) | Band assignments |
| --- | --- |
| 682 | *ν*(C_a_-S) |
| 743 | *ν*(pyr breathing) |
| 969 | *ν*(C_c_-C_d_)_6,7_ |
| 1130 | *ν*(pyr half-ring) |
| 1174 | *ν*(pyr half-ring) |
| 1225 | *δ*(C_m_-H) |
| 1314 | *δ*(C_m_-H) |
| 1360/1370 | *ν*(pyr half-ring) |
| 1400 | *ν*(pyr quarter-ring) |
| 1496 | *ν*(C_α_-C_m_) |
| 1551 | *ν*(C*_β_*-C*_β_*) |
| 1582 | *ν*(C_α_-C_m_) |
| 1634 | *ν*(C_α_-C_m_) |

Reference

1. Hu S, Morris IK, Singh JP, Smith KM, Spiro T*G.* Complete assignment of cytochrome *c* resonance Raman spectra via enzymic reconstitution with isotopically labeled hemes. J Am Chem Soc 1993;115:12446–58. https://doi.org/10.1021/ja00079a028
